# Prevalence and predictors of overweight and obesity among young adults in Lephalale between 2021 and 2023, Limpopo province, South Africa

**DOI:** 10.4102/jphia.v17i1.1383

**Published:** 2026-01-30

**Authors:** Themba T. Sigudu, Thandiwe N. Mkhatshwa, Kotsedi D. Monyeki, Moloko Matshipi

**Affiliations:** 1 Department of Physiology and Environmental Health, Faculty of Science and Agriculture, University of Limpopo, Sovenga, South Africa; 2 Department of Health and Society, Faculty of Health Sciences, University of the Witwatersrand, Johannesburg, South Africa

**Keywords:** overweight, obesity, young adults, rural health, diet, physical activity, smoking, alcohol consumption, South Africa

## Abstract

**Background:**

Overweight and obesity are rising health concerns in South Africa, increasingly affecting young adults in rural communities, and are influenced by distinct demographic, socio-economic and behavioural factors.

**Aim:**

This study aimed to determine the prevalence of body mass index (BMI)-defined overweight and obesity among young adults aged 18–29 years.

**Setting:**

The study was conducted in Lephalale, a predominantly rural-industrial area located within the Waterberg District Municipality of Limpopo province, South Africa.

**Methods:**

A community-based cross-sectional survey of 1063 adults aged 18–29 years from 42 rural settlements collected data on socio-demographic characteristics, employment, income, government grant receipt, physical activity (International Physical Activity Questionnaire – IPAQ), dietary practices (processed and fast food intake, fruit and vegetable consumption), smoking and alcohol use. BMI was calculated as weight divided by height squared (kg/m^2^) and classified using WHO criteria. Univariate and multivariable logistic regression analyses identified the predictors of combined overweight and obesity compared with normal or underweight participants.

**Results:**

The combined prevalence of overweight and obesity was 35%. Significant independent predictors included age 25–29 years (adjusted odds ratio [AOR]: 2.05; 95% confidence interval [CI]: 1.40–3.00), government grant receipt (AOR: 1.55; 95% CI: 1.05–2.28), daily processed food intake (AOR: 1.85; 95% CI: 1.25–2.73), weekly fast food intake (AOR: 1.70; 95% CI: 1.18–2.45), smoking (AOR: 1.25; 95% CI: 1.00–1.72) and daily alcohol use (AOR: 2.10; 95% CI: 1.35–3.15). Overweight and obesity were prevalent among rural young adults and were associated with socio-economic vulnerability and modifiable lifestyle behaviours.

**Conclusion:**

Overweight and obesity were highly prevalent among young adults living in a rural-industrial setting in Limpopo province.

**Contribution:**

The findings highlight priority behavioural and social determinants that can inform targeted public health interventions and obesity-prevention policies in transitioning rural communities.

## Introduction

Overweight and obesity have emerged as major global public health concerns, with their prevalence having nearly tripled since 1975.^1^ These conditions significantly contribute to noncommunicable diseases (NCDs), such as cardiovascular disease, type 2 diabetes, hypertension and certain cancers, collectively accounting for over 70% of global deaths each year.^2,3^ Beyond mortality, obesity imposes major economic costs through lost productivity and rising healthcare expenditure.^4^

Initially, obesity was largely confined to high-income countries; however, with rapid urbanisation, economic development and globalisation of food systems, it has caused an ‘obesogenic transition’ in low- and middle-income countries.^5,6^ This transition has resulted in increased consumption of processed foods high in sugar, salt and fat, coupled with reduced physical activity.^7,8^ In sub-Saharan Africa, obesity is now recognised as a growing epidemic affecting both urban and rural populations.^9^

South Africa is among the countries with the highest obesity rates in Africa, with adult prevalence estimated at 41% among women and 11% among men.^10^ National survey data have further highlighted widening socio-economic and gender disparities in overweight and obesity.^11^ Contributing factors include urbanisation, food insecurity, limited access to affordable healthy foods and inadequate opportunities for physical activity.^12,13^ These patterns pose major challenges to the national health-system response and broader social-determinant environment.

Despite the availability of national data, limited empirical evidence exists for young adults in rural contexts, whose behavioural and socio-economic realities differ substantially from those of urban peers. Rural populations in South Africa often experience persistent poverty, high unemployment and limited access to healthcare and recreational facilities.^5,13^ Such conditions encourage dependence on inexpensive, energy-dense foods and discourage physically active lifestyles, increasing vulnerability to obesity and related disorders.

Lephalale in Limpopo province represents this rural–industrial transition. The area is undergoing rapid mining-driven development amidst persistent structural poverty. Young adulthood (18–29 years) represents a pivotal life stage when health behaviours, including dietary habits, alcohol consumption and physical activity, are established and often persist into later life.^14,15^ Understanding the magnitude and determinants of overweight and obesity in this demographic is essential for designing targeted, locally relevant interventions.

Previous studies from the broader Ellisras region have primarily examined obesity and cardiometabolic risk among children and adolescents,^16^ yet contemporary data on young adults remain scarce. Generating updated, context-specific evidence is therefore critical to guide local prevention strategies aligned with South Africa’s National Strategy for the Prevention and Control of Obesity.^17^

Accordingly, this study estimated the prevalence of BMI-defined overweight and obesity and identified independent sociodemographic, economic and behavioural predictors among young adults in Lephalale, Limpopo province, South Africa (2021–2023).

## Research methods and design

### Study design and population

A community-based cross-sectional study was conducted between 2021 and 2023 among young adults aged 18–29 years from 42 rural settlements of Lephalale, Limpopo province, South Africa. The study captured a representative sample of adults living in the area during the study period to describe current weight-status patterns and associated factors. Although data collection has been performed in three annual rounds, the dataset represents a single pooled cross-sectional analysis rather than a longitudinal follow-up.

### Study setting

The study was conducted in the rural areas of Lephalale, located in the northwestern part of the Waterberg District Municipality in Limpopo province, South Africa, lying between 23°30′ and 24°00′ South latitude and 27°30′ and 28°00′ East longitude, bordered by the Blouberg, Modimolle, Mogalakwena and Thabazimbi municipalities and situated near the international border with Botswana. The rural areas of Ellisras comprise 42 settlements, home to an estimated 50 000 people, located approximately 70 km from the town of Ellisras. The region’s economy is mainly driven by mining, contributing 59.21% to its gross domestic product (GDP), while other key sectors include agriculture, manufacturing and electricity, with the latter accounting for 11.33% of the GDP and 69.65% of the electricity production in the Waterberg area. A large portion of the population is engaged in subsistence farming and cattle farming, with a smaller segment working in education and government services. Despite these economic activities, the area faces challenges such as high unemployment, poverty and low life expectancy, significantly affecting the rural communities.

### Inclusion criteria

Participants were included in the study if they were between 18 years and 29 years of age, had been residing in the study area for at least 6 months prior to enrolment, and provided written informed consent to participate. Data for individuals who were unable to give informed consent or had incomplete anthropometric data were excluded from the analysis.

### Data collection

Data were collected through interviewer-administered questionnaires adapted from validated national health survey instruments. Sociodemographic variables included age, sex, employment status (employed, unemployed or student), monthly household income (≤ R5000.00; R5001.00 – R10 000.00; or > R10 000.00), and government grant receipt (yes or no).

Behavioural variables comprised several domains. Physical activity was assessed using the International Physical Activity Questionnaire (IPAQ-long form), which covered work, leisure and travel-related activities. Based on total metabolic equivalent minutes per week (MET-min/week), participants were categorised as having low (< 600 MET-min/week), moderate (600 MET-min/week – 3000 MET-min/week) or high (> 3000 MET-min/week) activity levels.

Dietary habits were evaluated by the frequency of processed food, fast food, and fruit and vegetable consumption, classified as daily, weekly, or rarely and never. For this study, processed food referred to packaged or refined items such as sausages and sweetened snacks, while fast food referred to commercially prepared quick-service meals.

Smoking status was recorded as current smoker (yes or no), and alcohol use was assessed by reported frequency, categorised as daily, weekly or rarely and never.

### Anthropometric measurements

Anthropometric measurements were performed according to World Health Organization (WHO) standards using calibrated digital scales and stadiometers. Height and weight were taken to the nearest 0.1 cm and 0.1 kg, respectively, with participants wearing light clothing and no shoes. Body mass index (BMI) was calculated as weight in kilograms divided by height in metres squared (kg/m^2^) and classified into four standard categories: underweight (< 18.5 kg/m^2^), normal weight (18.5 kg/m^2^ – 24.9 kg/m^2^), overweight (25.0 kg/m^2^ – 29.9 kg/m^2^) and obese (≥ 30.0 kg/m^2^). In addition, waist and hip circumferences as well as skinfold thicknesses were measured following the International Society for the Advancement of Kinanthropometry (ISAK) protocols to provide supplementary indicators of adiposity.

### Data analysis

Data were analysed using Stata or SE 21. Descriptive statistics (means, frequencies and percentages) summarised participant characteristics.

Univariate logistic regression identified factors associated with overweight and obesity (BMI ≥ 25 kg/m^2^). Variables with *p* < 0.10 were entered into multivariable logistic regression using forward stepwise selection. Results are presented as odds ratios (ORs) and adjusted odds ratios (AORs) with 95% confidence intervals (CIs); *p* < 0.05 denoted significance.

### Ethical considerations

Ethical clearance to conduct this study was obtained from the University of Limpopo Turfloop Research Ethics Committee (No. TREC/323/2017:IR). The research adhered to established ethical principles to safeguard participants’ rights, dignity and wellbeing. Written informed consent was obtained from all participants before enrolment. Participant confidentiality was maintained through secure and anonymised data handling procedures. The study was designed with cultural sensitivity, ensured minimal risk, and sought to contribute positively to community health through improved child nutrition outcomes. Engagement with local stakeholders was maintained throughout the research process to enhance contextual relevance, promote fairness and facilitate equitable dissemination of results. The investigators ensured transparency, avoided bias and declared any potential conflicts of interest to maintain scientific integrity and accountability.

## Results

### Participants’ characteristics

Table 1 shows the socio-demographic characteristics of participants enrolled in the Lephalale study (2021–2023). A total of 1063 young adults aged 18–29 years participated in the study, comprising 635 males (59.7%) and 428 females (40.3%). The largest age group was 20–24 years, representing nearly half of the sample (47.0%), followed by those aged 18–19 years (32.9%) and 25–29 years (20.0%). The age distribution was similar between males and females, indicating a balanced representation across the three categories.

**TABLE 1:** Sociodemographic characteristics of participants (Lephalale 2021–2023).

Characteristic	Category	Male		Female		Total	
		*n*	%	*n*	%	*n*	%
Age (years)	18–19	200	31.5	150	35.3	350	32.9
	20–24	300	47.2	200	47.1	500	47.0
	25–29	135	21.3	78	18.4	213	20.0
Employment	Employed	240	37.8	160	37.6	400	37.6
	Unemployed	230	36.2	170	40.0	400	37.6
	Student	165	26.0	98	23.1	263	24.7
Household income (R)	≤ 5000.00	190	29.9	160	37.6	350	32.9
	5001.00–10 000.00	270	42.5	180	42.4	450	42.3
	> 10 000.00	175	27.5	88	20.7	263	24.7
Government grant	Yes	300	47.2	250	58.8	550	51.7

R, South African rand.

In terms of employment status, approximately equal proportions of males and females were employed (37.8% vs. 37.6%), while unemployment rates were slightly higher among females (40.0%) than males (36.2%). Students comprised about one-quarter of participants (24.7%), with a marginally greater proportion of males (26.0%) than females (23.1%).

Household income levels showed the majority of participants (42.3%) falling within the R5001.00 – R10 000.00 range, while 32.9% reported earning ≤ R5000.00 and 24.7% above R10 000.00. Notably, low-income status (≤ R5000.00) was more common among females (37.6%) than males (29.9%), exhibiting persistent gender-based economic disparities.

More than half of participants (51.7%) reported receiving government grants, with a higher prevalence among females (58.8%) compared with males (47.2%).

### Lifestyle behaviours

Table 2 presents the distribution of behavioural factors and dietary patterns among study participants in Lephalale (2021–2023). Regarding processed food consumption, 37.6% of participants reported daily intake, 37.6% weekly, and 24.7% rarely or never. Daily consumption was more common among females (42.4%) than males (34.6%), suggesting higher exposure to energy-dense and processed foods among women. Similarly, frequent fast food consumption was prevalent, with 32.9% of participants eating fast food daily and 42.3% weekly. A larger proportion of females (47.1%) than males (39.4%) reported weekly fast food intake, indicating greater consumption of commercially prepared foods among women.

**TABLE 2:** Behavioural factors and dietary patterns of participants (Lephalale 2021–2023).

Variable	Category	Male		Female		Total	
		*n*	%	*n*	%	*n*	%
Processed food	Daily	220	34.6	180	42.4	400	37.6
	Weekly	250	39.3	150	35.3	400	37.6
	Rare or never	165	26.0	98	23.1	263	24.7
Fast food	Daily	210	33.0	140	32.9	350	32.9
	Weekly	250	39.4	200	47.1	450	42.3
	Rare or never	175	27.6	88	20.7	263	24.7
Fruits or vegetables	Daily	250	39.4	200	46.7	450	42.3
	Weekly	230	36.2	170	40.0	400	37.6
	Rare or never	135	21.3	78	18.3	213	20.0
Smoking	Yes	200	31.5	63	14.7	263	24.7
Alcohol use	Daily	180	28.3	70	16.4	250	23.5
	Weekly	240	37.8	160	37.4	400	37.6
	Rare or never	215	33.9	198	46.3	413	38.8

In contrast, fruit and vegetable intake was more favourable, with 42.3% consuming them daily and 37.6% weekly. Daily consumption was slightly higher among females (46.7%) than males (39.4%), indicating better adherence to recommended dietary practices among women. However, one-fifth of participants (20.0%) reported rarely or never consuming fruits or vegetables, highlighting persistent dietary imbalances.

Substantial gender differences were observed in smoking and alcohol use. Smoking was reported by nearly one-third of males (31.5%) but only 14.7% of females, resulting in an overall prevalence of 24.7%. Similarly, daily alcohol consumption was higher among males (28.3%) compared with females (16.4%). While weekly alcohol use was similar between sexes (37.8% males vs. 37.4% females), a greater proportion of females reported rare or no alcohol use (46.3%) compared to males (33.9%).

### Body mass index distribution among participants

Table 3 presents the distribution of BMI categories among young adults in Lephalale from 2021 to 2023. The results showed that most participants (60.0%) had a BMI within the normal range (18.5 kg/m^2^ – 24.9 kg/m^2^), suggesting that the majority maintained a healthy weight status. However, a substantial proportion (35.0%) of participants were classified as overweight or obese, reflecting a notable burden of excess body weight in this rural–industrial setting.

**TABLE 3:** Body mass index distribution of participants (Lephalale, 2021–2023).

BMI category	Male		Female		Total	
	*n*	%	*n*	%	*n*	%
Underweight (< 18.5 kg/m^2^)	35	5.5	18	4.2	53	5.0
Normal weight (18.5 kg/m^2^ – 24.9 kg/m^2^)	385	60.6	255	59.6	640	60.0
Overweight (25.0 kg/m^2^ – 29.9 kg/m^2^)	125	19.7	88	20.6	213	20.0
Obese (≥ 30.0 kg/m^2^)	90	14.2	70	15.6	160	15.0

**Total**	**635**	**100**	**431**	**100**	**1066**	**100**

BMI, body mass index.

Among males, 19.7% were overweight and 14.2% obese, while among females, 20.6% were overweight and 15.6% obese. Although females exhibited slightly higher rates of both overweight and obesity, the sex differences were relatively modest. Conversely, underweight prevalence was low overall (5.0%) and slightly higher in males (5.5%) than in females (4.2%). The combined overweight and obesity prevalence was 35.0% among this young adult population.

### Univariate analysis

Table 4 presents the univariate logistic regression analysis of predictors correlated with overweight and obesity among young adults in Lephalale from 2021 to 2023. Age was a strong predictor: compared with participants aged 18–19 years (reference group), those aged 20–24 years had 1.45 times higher odds of being overweight or obese (OR = 1.45; 95% CI: 1.05–2.03; *p* = 0.02), while participants aged 25–29 years had more than double the odds (OR = 2.10; 95% CI: 1.45–3.00; *p* < 0.001). These findings indicate an age-related increase in the likelihood of excess body weight.

**TABLE 4:** Univariate predictors of overweight and obesity.

Predictor	Category	OR	95% CI	*p*-value
Age (years)	20–24	1.45	1.05–2.03	0.020
	25–29	2.10	1.45–3.00	< 0.001
Sex	Male	1.10	0.85–1.42	0.450
Employment	Employed	0.85	0.63–1.15	0.280
Income	Moderate	1.35	1.00–1.82	0.050
Government grant	Yes	1.60	1.10–2.30	0.010
Processed food (daily)	Yes	1.90	1.40–2.55	< 0.001
Fast food (weekly)	Yes	1.80	1.35–2.50	< 0.001
Fruit and veg (rare)	Yes	2.05	1.30–3.10	0.020
Smoking	Yes	1.40	1.05–1.86	0.030
Alcohol (daily)	Yes	2.20	1.45–3.35	< 0.001

OR, odds ratio; CI, confidence interval.

Sex and employment status were not significantly associated with overweight and obesity (*p* > 0.05). Although males showed slightly higher odds (OR = 1.10), the association was not statistically significant (*p* = 0.45). Similarly, employment was not a significant predictor (OR = 0.85; *p* = 0.28). Participants with moderate household income (R5001.00 – R10 000.00) had marginally higher odds of overweight and obesity (OR = 1.35; 95% CI: 1.00–1.82; *p* = 0.05), suggesting a borderline correlation between income and body weight.

Government grant recipients were more likely to be overweight or obese (OR = 1.60; 95% CI: 1.10–2.30; *p* = 0.01), implying a possible link between socio-economic vulnerability and unhealthy weight gain. Among dietary and lifestyle variables, several strong associations were observed. Both daily processed food consumption (OR = 1.90; 95% CI: 1.40–2.55; *p* < 0.001) and weekly fast food intake (OR = 1.80; 95% CI: 1.35–2.50; *p* < 0.001) were significant predictors, underlining the contribution of energy-dense diets to excess weight. Participants who rarely consumed fruits and vegetables had over twice the odds of being overweight or obese (OR = 2.05; 95% CI: 1.30–3.10; *p* = 0.02).

Smoking (OR = 1.40; 95% CI: 1.05–1.86; *p* = 0.03) and daily alcohol consumption (OR = 2.20; 95% CI: 1.45–3.35; *p* < 0.001) were also significantly correlated with higher odds of overweight and obesity.

### Multivariable analysis

Table 5 demonstrates the results of the multivariable logistic regression identifying independent predictors of overweight and obesity among young adults in Lephalale from 2021 to 2023. After adjusting for potential confounders, several factors remained significantly correlated with excess body weight.

**TABLE 5:** Multivariable logistic regression predictors of overweight and obesity.

Predictor	AOR	95 % CI	*p*-value
Age 25–29 years	2.05	1.40–3.00	< 0.001
Government grant (yes)	1.55	1.05–2.28	0.030
Processed food (daily)	1.85	1.25–2.73	0.002
Fast food (weekly)	1.70	1.18–2.45	0.004
Smoking (yes)	1.25	1.00–1.72	0.049
Alcohol (daily)	2.10	1.35–3.15	< 0.001

AOR, adjusted odds ratio; CI, confidence interval.

Age showed a strong independent effect: participants aged 25–29 years had more than twice the odds of being overweight or obese compared with those aged 18–19 years (AOR = 2.05; 95% CI: 1.40–3.00; *p* < 0.001). This finding confirms the progressive increase in overweight and obesity risk with advancing age, even within the young adult age range.

Socio-economic factors also played a role. Individuals receiving government grants had significantly higher odds of being overweight or obese (AOR = 1.55; 95% CI: 1.05–2.28; *p* = 0.03), suggesting that economic dependency or lower income may contribute to unhealthy dietary and lifestyle habits associated with weight gain.

Dietary patterns were strong and consistent predictors. Participants reporting daily consumption of processed foods had 1.85 times higher odds of being overweight or obese (AOR = 1.85; 95% CI: 1.25–2.73; *p* = 0.002), while those consuming fast food weekly had 1.70 times higher odds (AOR = 1.70; 95% CI: 1.18–2.45; *p* = 0.004).

Behavioural factors, including smoking and alcohol use, also remained significant in the adjusted model. Smokers were 1.25 times more likely to be overweight or obese compared with nonsmokers (AOR = 1.25; 95% CI: 1.00–1.72; *p* = 0.049), although this association was modest. Daily alcohol consumption showed the strongest behavioural effect, with drinkers exhibiting more than double the odds of overweight and obesity (AOR = 2.10; 95% CI: 1.35–3.15; *p* < 0.001).

## Discussion

This study examined the prevalence and determinants of overweight and obesity among young adults in Lephalale, Limpopo province, South Africa. The findings revealed a considerable burden of excess body weight in this rural–industrial population. Although most participants had body mass indices within the normal range, a substantial proportion were classified as overweight or obese. This pattern aligns with national and regional trends indicating a growing shift towards diet-related and lifestyle-related health risks among younger populations in South Africa.^18,19^

The sociodemographic profile reflected a predominance of economically active young adults with moderate employment levels and widespread reliance on government support. These characteristics suggest socio-economic vulnerability typical of many rural and peri-industrial settings in South Africa. The observed association between receipt of government grants and overweight or obesity implies that income dependency, limited access to affordable healthy foods, and reliance on inexpensive, energy-dense options may collectively contribute to unhealthy weight gain.^20,21^ Similar trends have been reported in other African and low- to middle-income contexts, where economic and nutritional transitions co-exist.^22,23^

The study demonstrated strong associations between dietary behaviours and overweight or obesity. Frequent consumption of processed and fast foods emerged as an important predictor, consistent with global evidence linking energy-dense, nutrient-poor diets to increased adiposity.^24,25,26^ Participants reporting higher intake of these foods were significantly more likely to have excess body weight, supporting the growing evidence that rural populations are now experiencing similar dietary shifts to those previously confined to urban settings.^27,28^

Although fruit and vegetable intake was relatively common, a notable proportion of participants reported infrequent consumption, reflecting the persistence of unbalanced dietary patterns. This co-existence of high processed food intake and inadequate fruit and vegetable consumption indicates the ongoing ‘nutrition transition’, characterised by the displacement of traditional diets by refined and commercially prepared foods.^29,30^

Behavioural risk factors also played a significant role. Both smoking and alcohol use were associated with overweight and obesity, although the direction and magnitude of these effects vary across populations. The association between alcohol intake and excess body weight observed in this study supports prior research linking regular alcohol consumption to increased caloric intake and altered lipid metabolism.^31,32^ The modest positive association between smoking and overweight may reflect the clustering of multiple lifestyle risk factors, including unhealthy diet and low physical activity, rather than a direct causal effect.^33^

Age emerged as a strong independent determinant of overweight and obesity, with older young adults more likely to exhibit higher body mass indices than their younger counterparts. This pattern mirrors findings from other South African and international studies showing that early adulthood represents a critical life stage when lifestyle behaviours consolidate and long-term metabolic trajectories are established.^34,35,36^ The results are consistent with longitudinal findings from the Ellisras cohort, which documented progressive increases in body mass and adiposity from adolescence through young adulthood.^37^

The findings highlight the shifting health landscape in rural and peri-industrial communities, where undernutrition and overweight increasingly co-exist. Economic transitions, urban influence and changing food environments contribute to this dual burden of malnutrition.^19,38^ Addressing these challenges requires a multifaceted public-health approach encompassing improved nutrition education, community-based physical-activity initiatives and policies aimed at reducing access to ultraprocessed foods and harmful substances such as alcohol. Efforts to promote healthier diets should prioritise affordability and accessibility, particularly for low-income populations reliant on social support systems.

### Strengths and limitations of the study

A major strength of this study lies in its focus on young adults from a rural–industrial setting, a population group that has received limited empirical attention in South Africa. By targeting this demographic, the study contributes valuable, context-specific evidence on the early onset of overweight and obesity in transitioning communities. The use of validated and standardised measurement tools, such as the WHO protocols for anthropometry and the IPAQ for physical activity, enhanced the reliability and comparability of findings. The relatively large sample size and inclusion of both sexes across a defined age range further strengthen the representativeness of the results.

However, several limitations should be acknowledged. The cross-sectional design precludes causal inference between behavioural, socio-economic and anthropometric variables. Self-reported dietary and lifestyle information may have been affected by recall or social desirability bias. Additionally, the study did not include objective measures of physical activity or biochemical indicators such as lipid or glucose profiles, which could provide deeper insights into metabolic health. Despite these limitations, the study offers important baseline evidence that can inform longitudinal research and guide public health interventions aimed at preventing obesity among rural and peri-industrial young adults in South Africa.

## Conclusion

This study demonstrated that overweight and obesity are highly prevalent among young adults living in a rural–industrial setting in Lephalale, Limpopo province. The findings revealed that increasing age, socio-economic dependency and unhealthy lifestyle behaviours, including frequent consumption of processed and fast foods, smoking and daily alcohol use, were significant predictors of excess body weight. These results indicate that the nutrition and lifestyle transitions previously confined to urban areas are now increasingly affecting rural populations.

Targeted interventions promoting healthy dietary habits, reducing reliance on energy-dense processed foods, and addressing behavioural risks are urgently needed. Public health policies should prioritise young adults, integrating nutrition education, physical-activity promotion and community-based strategies to prevent early onset of obesity-related noncommunicable diseases. Strengthening local food systems and improving access to affordable, nutritious foods may further support long-term health improvement in transitioning rural communities.

### Recommendations

Public health programmes should prioritise early prevention of overweight and obesity during young adulthood by promoting balanced nutrition and active living. Community-based health education initiatives that emphasise moderation of processed and fast food intake, reduce alcohol consumption and increase fruit and vegetable intake are critical. Policies should aim to improve access to affordable, healthy foods through local agricultural support, food subsidies and school-based or community-based nutrition schemes.

Furthermore, multisectoral collaboration involving health, education and social-development departments is essential to integrate obesity prevention into existing social support systems, particularly for grant-dependent populations. Future interventions should also include regular monitoring and behavioural surveillance to track weight trends and evaluate programme effectiveness. Strengthening these preventive strategies can contribute to reducing the long-term burden of noncommunicable diseases and improving the health trajectories of rural and transitioning South African communities.
